# Contribution of −Omics Technologies in the Study of *Porphyromonas gingivalis* during Periodontitis Pathogenesis: A Minireview

**DOI:** 10.3390/ijms24010620

**Published:** 2022-12-30

**Authors:** Josefa Nuñez-Belmar, Mauricio Morales-Olavarria, Emiliano Vicencio, Rolando Vernal, Juan P. Cárdenas, Cristian Cortez

**Affiliations:** 1 Centro de Genómica y Bioinformática, Facultad de Ciencias, Ingeniería y Tecnología, Universidad Mayor, Santiago 8580745, Chile; 2 Escuela de Tecnología Médica, Facultad de Ciencias, Pontificia Universidad Católica de Valparaíso, Valparaíso 2373223, Chile; 3 Periodontal Biology Laboratory, Faculty of Dentistry, Universidad de Chile, Santiago 8380492, Chile; 4 Escuela de Biotecnología, Facultad de Ciencias, Ingeniería y Tecnología, Universidad Mayor, Santiago 8580745, Chile

**Keywords:** periodontitis, microbial dysbiosis, *Porphyromonas gingivalis*, keystone pathogen, immunoinflammatory response, metagenomics, metatranscriptomics, omics technologies

## Abstract

Periodontitis is a non-communicable chronic inflammatory disease characterized by the progressive and irreversible breakdown of the soft periodontal tissues and resorption of teeth-supporting alveolar bone. The etiology of periodontitis involves dysbiotic shifts in the diversity of microbial communities inhabiting the subgingival crevice, which is dominated by anaerobic Gram-negative bacteria, including *Porphyromonas gingivalis*. Indeed, *P. gingivalis* is a keystone pathogen with a repertoire of attributes that allow it to colonize periodontal tissues and influence the metabolism, growth rate, and virulence of other periodontal bacteria. The pathogenic potential of *P. gingivalis* has been traditionally analyzed using classical biochemical and molecular approaches. However, the arrival of new techniques, such as whole-genome sequencing, metagenomics, metatranscriptomics, proteomics, and metabolomics, allowed the generation of high-throughput data, offering a suitable option for bacterial analysis, allowing a deeper understanding of the pathogenic properties of *P. gingivalis* and its interaction with the host. In the present review, we revise the use of the different −omics technologies and techniques used to analyze bacteria and discuss their potential in studying the pathogenic potential of *P. gingivalis*.

## 1. Introduction

Periodontitis is a non-communicable chronic inflammatory disease characterized by the progressive and irreversible destruction of the periodontium [1,2]. Periodontitis is a long-term disease with a high prevalence worldwide, being the most prevalent form of osteolytic pathology in humans [3,4]. The etiology of periodontitis lies in the constant challenge of the dysbiotic biofilm attached to the tooth in the subgingival crevice, which leads to a deregulated host immune response responsible for the clinical phenotype of the disease [5].

**Figure 1:**
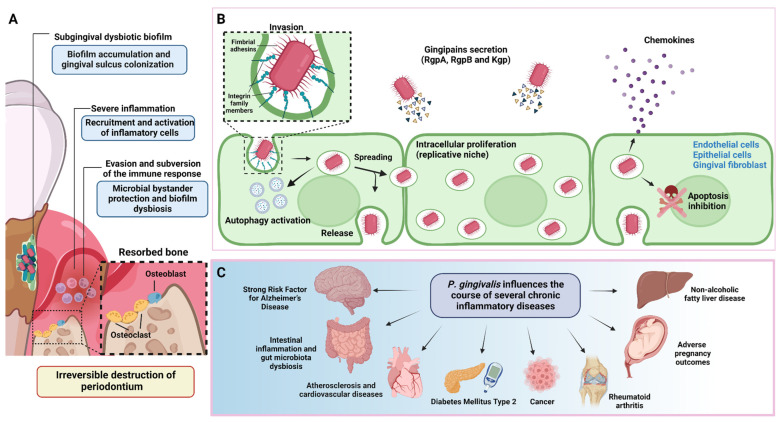
Periodontitis pathogenesis and *P. gingivalis* overview. (**A**) During the onset and progression of periodontitis, *P. gingivalis* resides in the subgingival biofilm adhered to the tooth surface, where it interacts metabolically with other bacteria, inducing them to express different virulence factors with pathogenic potential. In parallel, *P. gingivalis* acts as a keystone pathogen, altering the regulation of the immune response in the susceptible host. The metabolic synergism and immune response subversion provide the nutritional and protective conditions required by the dysbiotic subgingival community to increase their diversity and abundance, with the concomitant induction of a strong, destructive inflammatory response. Together, all these activities cause irreversible connective tissue breakdown and resorption of the tooth-supporting alveolar bone, the critical hallmark of periodontitis that causes tooth loss. (**B**) To invade the periodontium, *P. gingivalis* uses various virulence factors that allow it to colonize, replicate, and spread in different subsets of cells to increase its progeny and generate infection. (**C**) In addition to causing tooth loss, *P. gingivalis*-induced periodontitis can also affect systemic health, influencing the course of other diseases and conditions. This figure was created using BioRender.com.

*Porphyromonas gingivalis* is a well-characterized member of the dysbiotic subgingival microbiota and a key bacterium in the aetiopathogenesis of periodontitis [6,7]. It is an anaerobe, asaccharolytic, non-motile, Gram-negative bacterium associated with destructive chronic periodontitis [8]. Its association with periodontitis is based fundamentally on its virulence properties, recurrent presence in affected sites, and higher serum antibody levels in patients diagnosed with chronic periodontitis. In addition, periodontal health indicators are inversely correlated with the presence of this periodontal pathogen [8,9,10]. Moreover, periodontitis induced by *P. gingivalis* may influence the course and pathogenesis of other chronic inflammatory disorders [11]. *P. gingivalis* expresses several virulence factors that enable it to colonize, invade, and disrupt periodontal tissues [8,12]. Despite its low-relative abundance in the subgingival biofilm, *P. gingivalis* operates as a keystone pathogen, being critical in promoting the host inflammatory and osteolytic response involved in periodontitis onset and progression [7,13,14]. Furthermore, *P. gingivalis* significantly changes the bacterial taxa that comprise symbiotic microbial communities for heterotypical communities with recognized destructive inflammatory potential [6,7] (Figure 1). *P. gingivalis* strain virulence varies among different types, and this diversity may alter its keystone pathogen capacity [14,15,16]. Consequently, precise analysis methods are required for a better understanding of *P. gingivalis* in the context of its different pathogenic potential and its modulating effect on multispecies heterotopic communities in the periodontitis-related biofilm.

Omics technologies have revolutionized modern biology, enabling simultaneous analysis of multiple molecular components in response to environmental stimuli across time [17]. These technological approaches, mainly metagenomics, metatranscriptomics, proteomics, and metabolomics, have the potential to increase the efficiency of information to elucidate complex systems [18] and have been used to understand the composition and function of the oral microbiome during health and disease [19,20,21]. Hence, in the present minireview, we detail the different −omics technologies currently used to carry out microbiological studies and discuss their utility in assessing the ability of *P. gingivalis* to interact with other bacteria and cause periodontitis-related damage.

## 2. A Brief Description of the −Omics Disciplines

**Figure 2:**
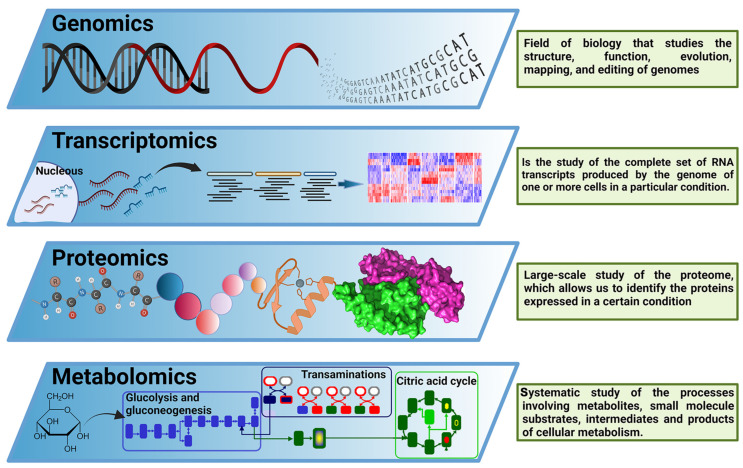
The central dogma of −omics technologies. Overview of −omics methodological approaches most relevant for biological and biomedical research. These include genomics, transcriptomics, proteomics, and metabolomics, covering the main components of the Central Dogma of M olecular B iology. This figure was created using BioRender.com.

In past decades, molecular biology had a reductionist approach to understanding biological systems, breaking down a complex problem into its constituent parts to solve them individually [22]. Since the complete sequencing of the first viral genomes, and especially since the sequencing of the first whole bacterial genome [23], the rise of genomics offered a new research paradigm focused on the analysis of a big amount, or even the totality, of the genes in one organism. Later, the “−omics” term was implemented as a neologism related to the study of different cellular components. As a multidisciplinary field, the “−omics” disciplines comprise a set of techniques and technologies involving the analysis of biological components such as nucleic acids, epigenetic signatures, proteins, or metabolites (among other components), in a high-throughput and untargeted manner, enabling simultaneous analysis of multiple molecular components in response to environmental stimuli at a given time [22]. These new techniques are focused on the massive study of genes (genomics), transcripts (transcriptomics), proteins (proteomics), and metabolites (metabolomics), among others, covering every aspect of the so-called Central Dogma of Molecular Biology (Figure 2). Additionally, those techniques can be focused on analyzing complex samples (from environmental or host-associated niches) without previous culture isolation processes, configuring the “meta− −omics” techniques. Therefore, (meta)-genomics analyzes genetic material recovered from an organism, communities of microorganisms, or samples from a specific environment [24], including both protein-encoding genes and non-coding RNA genes [25]. It is worth remembering that experiments focused on the deep sequencing of marker-targeted amplicons, such as different 16S rRNA variable regions [26], cannot be considered metagenomic studies since these experiments do not analyze the genetic composition of the organisms present in the sample but are a proxy of it. However, deep amplicon sequencing experiments are legit examples of the power of next-generation sequencing for improving microbial communities research [26]. Therefore, this minireview also includes a few relevant deep amplicon sequencing studies.

(Meta)-transcriptomics is the analysis of RNA molecules, such as messenger RNA (mRNA), ribosomal RNA (rRNA), transfer RNA (tRNA), and other non-coding RNAs produced by an organism, population of organisms, or product of the interaction between organisms and the environment [27]. (Meta)-proteomics is the analysis of proteins produced by an organism or population of organisms and their function; and finally, metabolomics refers to the complete set of small chemical compounds, such as metabolic intermediates, hormones, secondary metabolites, and other signaling molecules to be found within a biological sample [28].

**Figure 3:**
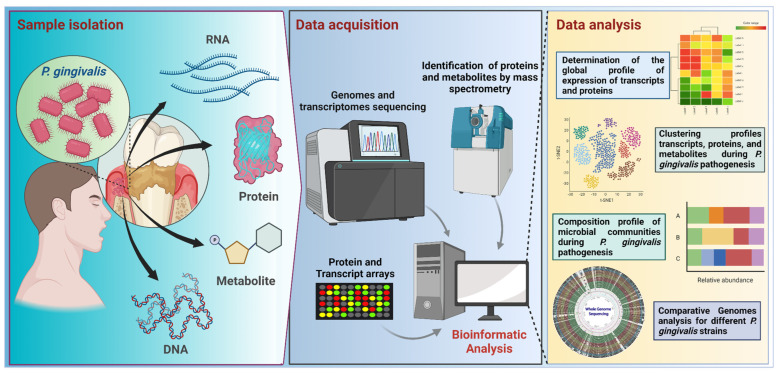
Workflow for −omic research focused on *P. gingivalis* studies. Samples from subgingival plaque are used for metagenomic, metatranscriptomic, proteomic, and metabolomic analyses. Samples are processed in the data acquisition stage by using sequencing or microarray techniques for DNA and RNA, or by mass spectrometry, used to identify proteins and metabolites. Bioinformatic workflows involve quality control and several downstream analyses, such as data clustering or data classification. For example, in the case of sequencing-based procedures, the analyses start with nucleic acid extraction, purification, quality control, library preparation, and sequencing; raw data from sequencing is analyzed using bioinformatics approaches, depending on the target or strategy for sequencing, such as a marker-targeted amplicon (e.g., a region of the 16S rRNA gene) or a shotgun sequencing (for a whole metagenome). In the case of isolated genome sequencing, generated data can be used in comparative genomic analysis. Transcriptomics studies are directed to RNA samples, involving RNA extraction, isolation, and quality checking, before sequencing. Most transcriptome studies are focused on mRNAs, focusing on the identification of the upregulated and downregulated gene expression. The further analysis comprises the determination of the global expression profile, clustering profile, and community composition profile. This figure was created using BioRender.com.

All these technologies have revolutionized and positively impacted biological sciences since they allow an increase in the non-targeted simultaneous detection, quantification, and monitoring of a large number of molecules present in a specific sample, optimizing the functional evaluation of the different biological systems. Some of those impacts have been observed in the field of *P. gingivalis* research, accumulating several studies in metagenomics, deep amplicon sequencing (sometimes miscalled as metagenomics), transcriptomics, proteomics, and metabolomics, as well as their -meta counterparts (Figure 3, Table 1, Table 2 and Table 3).

## 3. Comparative Genomic Studies

As an early “−omic” approach, genome sequencing and comparative genomics, using different clinical *P. gingivalis* isolates, have been powerful tools for understanding how genetic variability and pathogenicity are connected [29]. One of the most researched aspects of *P. gingivalis* comparative genomics is the relationship between strain gene content and the ability to produce periodontitis and its severity. Early studies, using microarrays [30] and comparative genomics [31], compared the genomic contents between strains ATCC^®^33277™ (considered a low-virulence variant) and W83 (highly virulent), suggesting the presence of highly divergent genes and extensive genomic rearrangements; those studies also found a set of strain-specific genes, such as transposable elements may be involved in the rise of several strain-specific genes. In the W83 strain, a polysaccharide capsule biosynthesis gene cluster was found as a potential feature that differentiates it from the ATCC^®^33277™ strain. Another study [32] also compared the invasive strain W83 with a non-invasive strain (AJW4), using whole-genome microarrays, suggesting a set of highly divergent genes encoding surface proteins, lipoproteins, capsular polysaccharide biosynthesis enzymes, regulatory and immunoreactive proteins, integrases, and transposases (Table 1).

**Table 1:** Summary of deep amplicon sequencing, comparative genomics and metagenomics research studies associated to this review.

Strategy	Method and Sample	Findings/Contributions	Reference
Deep amplicon sequencing	Sequencing and identification of 16S rRNA of patient’s subgingival plaque.	There is a strong correlation between bacterial community structure and disease status, that are present through functional genes and metabolic pathways.	[19]
Deep amplicon sequencing	Sequencing 16S rRNA and functional gene array. Subgingival dental plaque from healthy and periodontitis patients.	Differential microbial community composition and structure and the changes in the functional genes.	[33]
Deep amplicon sequencing	454-pyrosequencing of 16S rRNA gene libraries of patient’s subgingival plaque.	Definition of the core subgingival microbiome in health and periodontitis in the Chilean population.	[34]
Comparative genomics	Microarray to compare the total gene content of *P. gingivalis* strains W83 and ATCC^®^33277™.	Both strains exhibit 93% of the predicted genes in common; the other 7% showed low signal in ATCC^®^33277™.	[30]
Comparative genomics	Sequencing of the whole genome of *P. gingivalis* ATCC^®^33277™ and W83 strains.	Determination of the whole genome sequence of *P. gingivalis*. Compared to the W83 strain (more virulent), ATCC^®^33277™ showed extensive gene rearrangements.	[31]
Comparative genomics	Microarray to compare *P. gingivalis* W83 strain (invasive) and *P. gingivalis* AJW4 strain (non-invasive).	Identification of 125 open reading frames (ORF) that were polymorphic between both strains, suggesting internal variability among conserved genes.	[32]
Comparative genomics	Phylogenetics Analysis and comparative genomics of 32 *Porphyromonas* publicly available genomes (18 were canine oral isolates and 14 non-canine isolates).	Interspecies differences in the iron management of *Porphyromonas cangingivalis* and other species (including *P. gingivalis*) are associated with the presence of genes involved in heme biosynthesis	[35]
Comparative genomics	Comparative genomics analysis through 16S rRNA of 13 *Porphyromonas gingivalis* publicly available strains.	Strains evolutionary closest are W83 to W50 and HG66 to ATCC^®^33277™. From a genome-wide comparison analysis, the overall biological functions were similar between W83 and W50 strains; and between HG66 and ATCC^®^33277™.	[36]
Comparative genomics	Comparative genomics analysis through 16S rRNA of 19 *Porphyromonas gingivalis* publicly available strains.	The phylogeny showed that ATCC^®^33277™, and HG66 are evolutionary close and the closeness of W83, W50, and A7436. Identification of 1307 core/shared proteins and some functions that are unique or missing in individual *P. gingivalis* strains.	[37]
Comparative genomics	Comparative genomic analysis of 64 genomes *P. gingivalis* (62 from NCBI plus two clinical isolates CP3 and H3, from patients with severe periodontitis and periodontally healthy subject, respectively).	Differences in the genetic content between CP3 and H3 isolate. Compared to the rest of strains in NCBI, the two main strains in study showed variability in hemagglutinin genes, in vitro assays shows that the presence of this genes could be related with the virulence.	[38]
Comparative genomics	Comparative genomic of the red complex’s members: 18 strains of *T. forsythia*, 5 of *P. gingivalis*, and 14 of *T. denticola*.	Description of potential genetic cooperative interactions between members of the red complex, including metabolic complementations in fatty acid biosynthesis and virulence factor interactions.	[39]

The use of a higher number of genomes allows for establishing more features about *P. gingivalis* genetic variability, such as the definition of the pan-genome (i.e., the complete gene patrimony of the species), as well as the calculation of the core and accessory genomes (e.g., determining which genes are common to all genomes, or which genes are infrequent or unique among different strains). For example, a previous study using a genome set from 30 different clinical isolates obtained in human and canine samples [35] showed that *P. gingivalis* genomes lacked some genes involved in heme biosynthesis but possessed several genes involved in iron acquisition in comparison with other organisms such as *P. cangingivalis.* Another comparative genomic study using 13 strains [36] found a set of unique genes, including proteins involved in antimicrobial resistance, DNA repair, virulence, iron acquisition, and horizontal gene transfer. Another study [37], comparing 19 genomes, suggests the presence of two important groups of *P. gingivalis* genomes using a phylogenomic approach. Moreover, a relatively recent study [38] showed some patterns in the gene content between some highly virulent strains and others with low virulence. When comparing 64 genomes, this study reported that the core genome (i.e., all those genes that are detected in all the genomes) represents approximately 60 to 70% of the whole genetic content among *P. gingivalis* strains. This range of core genome content is similar to the observed in other pathogenic species, such as *Escherichia coli* and *Pseudomonas aeruginosa* [40]. The comparison of two Chilean clinical strains, termed CP3 and H3, isolated from a diseased and a healthy subject, respectively [38], showed a remarkable variability in the hemagglutinin content, differing in copy number (*hagA*/*hagC*) and in vitro activity. Additionally, it was found that whereas strain CP3 contained fimbriae type IV-encoding genes (associated with epithelial cell invasion), H3 strain genome encoded fimbria type I-associated genes (found in the healthy population). This study also showed a comparison with a total of 64 strains, exhibiting patterns of convergent evolution for different virulence factor conservation among *P. gingivalis* strains.

Pangenome analysis can be useful in understanding the relationship between *P. gingivalis* and other microorganisms from the so-called “red complex” [39]. For example, a comparative study between genomes of *P. gingivalis*, *Treponema denticola*, and *Tannerella forsythia* showed that, in addition to exhibiting an open pangenome, *P. gingivalis* might encode enzymatic functions that allow cross-feeding with other members of this complex. This synergism could explain how these organisms combine their functions to increase the severity of periodontitis.

## 4. Virulence Factors Expression and Environmental Adaptation Studies

As previously mentioned, metagenomics focuses on the sequencing of environmental DNA as a representation of the microorganisms co-inhabiting a particular niche [41], allowing the analysis of taxonomic composition and function of complex microbiomes [42] without the need to cultivate them [43]. Additionally, the use of metagenomes confirmed the presence of *P. gingivalis* in diseased and healthy individuals, with greater relative abundance in periodontitis-affected patients [44]. Furthermore, the virulence factors identification from metagenomic and metatranscriptomic (Table 2) analysis contributed to the identification of genes involved in different activities related to *P. gingivalis* pathogenicity, such as biofilm formation, colonization and invasion, immune evasion, iron acquisition/transport, lipopolysaccharide (LPS), and flagellar synthesis, among others [44,45,46]. Recently, a comparative analysis between the metatranscriptome of periodontal pocket samples from periodontitis patients and the transcriptome of laboratory-grown strains showed significant differences in transcriptional profiles, suggesting an influence of the community in vivo interactions on the expression profiles [47]. These authors used RNA sequencing for transcriptomic analysis detecting 125 genes significantly upregulated and 166 significantly downregulated in in vivo conditions. The classification of these genes revealed protein translation and metabolism, cell adhesion, and pathogenesis functions. Among the higher upregulated genes appear several transporters, including the hmu gene cluster encoding proteins involved in the hemin-acquisition pathway, confirming the iron nutritional dependence that this species suffered during infection. Moreover, the genes encoding gingipains (*kgp*, *hagD*, *rgpA*, *rgpB*), the main secreted-virulence factors of *P. gingivalis*, were the most prominently upregulated genes in vivo and in vitro [47]. Although these findings confirm the relevance of gingipains, they also suggest that the infection-environmental conditions do not modulate its expression.

**Table 2:** Summary of Transcriptomics and metatranscriptomics research studies associated to this review.

Strategy	Method and Sample	Findings/Contributions	Reference
Transcriptomics + metatranscriptomics	Comparison of virulence factors-expression profiles between clinical and culture isolates using RNA-seq.	Genes encoding virulence factors involved in protein degradation, iron uptake, and mobility of *P. gingivalis* were synergistically modulated by the presence of *F. nucleatum* and *T. denticola*.	[47]
Transcriptomics	Comparison of expression profiles between cultured *P. gingivalis* (strain W50) in biofilm and planktonic conditions using microarrays.	*P. gingivalis* grown in biofilm evidenced 18% differentially expressed genes when compared to bacteria grown planktonically.	[48]
Transcriptomics	Comparison of expression profiles between cultured *P. gingivalis* (ATCC^®^33277™) in biofilm and planktonic conditions using microarrays.	Compared to its planktonic state, *P. gingivalis* forming biofilm showed 92 differentially expressed genes related to: cell envelope, transport, binding of outer membrane proteins, transposases and oxidative stress.	[49]
Transcriptomics	Differential expression levels to assess sensitivity and survival of *P. gingivalis* (W83 strain) cultured in the presence of H2O2. Analyzes were performed using microarrays.	*P. gingivalis* changes its transcriptome in response to H_2_O_2_-induced oxidative stress. Ten minutes after exposure, genes related to DNA damage and repair increased their expression. After 15 min, genes related to protein fate, protein folding, and stabilization were upregulated.	[50]
Transcriptomics	Differential expression levels of *P. gingivalis* (W83 strain) cultured in the presence of polyphosphate (polyP75). Analyzes were performed using microarrays.	349 upregulated genes and 357 downregulated were identified. The exposure of *P. gingivalis* to polyP75 results in a perturbation of energy metabolism, cell envelope biosynthesis, and cell division.	[51]
Transcriptomics	Microarray analysis of *P. gingivalis* strain ATCC^®^33277™ expression profile when it is co-cultured with HEp-2 (epithelial cells) cells.	HEp-2 cells induced in *P. gingivalis* the upregulation of genes involved in oxidative stress response and heat shock protein-encoding genes.	[52]
Transcriptomics	The microarray of human monocyte isolated from healthy males challenged in vitro with *P. gingivalis* and LPS.	This study reveals new candidate periodontitis-associated differentially expressed genes.	[53]
Transcriptomics	Sequencing of human monocyte-derived DCs (MoDCs) infected with Pg381 or its defined isogenic mutants.	*P. gingivalis* fimbriae was essential in disrupting the DC-mediated immune homeostatic pathway.	[54]
Transcriptomics	Sequencing of cultured *P. gingivalis* (WT, ATCC^®^33277™ and isogenic ∆luxS strain).	Validation of the role of LuxS in regulating hemin uptake and microcolony formation with other bacteria. Both activities related to quorum sensing.	[55]
Metatranscriptomics	Sequencing of sample from patient’s subgingival plaque (same samples as Yost et al., 2015 [46]). In silico computational identification of sRNAs and phylogenetic analysis.	Small RNA in silico analysis revealed differential expression of 12,097 sRNAs and identification of 20 Rfam sRNA families. Gene ontology of differentially expressed sRNA was related to: amino acid metabolism, ethanolamine catabolism, signal recognition particle-dependent cotranslational protein targeting to membrane, intron splicing, carbohydrate metabolism, control of plasmid copy number, and stress response.	[45]
Metatranscriptomics	Sequencing of sample from patient’s subgingival plaque.	Identification of metabolic changes in the microbial community associated with the initial stage of dysbiosis. The metatranscriptome of *P. gingivalis* evidenced several upregulated virulence factors.	[46]
Metatranscriptomics	Sequencing of polymicrobial biofilms composed of 7 oral bacteria (isolated from healthy patients) cultured in vitro in the presence of *P. gingivalis* strains ATCC^®^33277™ or W83.	*P. gingivalis* W83 showed a more significant effect on symbiotic species than *P. gingivalis* ATCC^®^33277™. In addition, the gene expression of metabolic pathways and quorum sensing of commensal oral species were significantly influenced by strain W83.	[56]
Metatranscriptomics	Sequencing of in vitro cultured polymicrobial biofilm*: A. naeslundii* (MG1), *L. casei* (ATCC^®^334™), *S. mitis* (ATCC^®^49456™), *V. parvula* (ATCC^®^17745™), and *F. nucleatum* (ATCC^®^10953™) pathogenic: *P. gingivalis* (ATCC^®^33277™) and *A. actinomycetemcomitans* (ATCC^®^33384™).	Demonstrated that the addition of periodontal pathogens to a healthy multispecies biofilm changes its gene expression profiles.	[57]

During periodontitis pathogenesis, *P. gingivalis* colonize and reside in the subgingival biofilm, invade the gingival junctional epithelium, disrupt and evade the host immunity, and promote periodontium breakdown [9]. Evidence indicates that *P. gingivalis* exhibits different gene expression patterns depending on whether it is found as planktonic bacteria or forming biofilms [48]. A comparative study evaluating gene expression using microarrays identified 92 differentially expressed genes in biofilm bacteria than in the planktonic state [49]. Furthermore, the *P. gingivalis* biofilm undergoes significant changes in its transcriptional response when exogenously stimulated [50,51]. The in vitro challenge with an antimicrobial compound specific for Gram-negative bacteria evidenced 349 upregulated genes and 357 down-regulated genes when transcriptomically analyzed [51]. Similarly, *P. gingivalis* biofilm under hydrogen peroxide-induced oxidative stress exhibits temporal adaptation in its transcriptional levels. Ten minutes after the stimulus, DNA damage and repair genes were differentially expressed, and the genes related to fate and folding proteins, fifteen minutes after stimulation [50].

The gingival epithelium is a mechanical barrier representing the first line of defense and the initial interaction site between *P. gingivalis* and the host tissues [58]. *P. gingivalis* modifies its transcriptional profiles upon contact with human epithelial cells [52]. Transcriptomic analysis revealed that epithelial cells differentially upregulated bacterial genes involved in response to oxidative stress and those encoding heat shock proteins. These genes are critical for resisting harsh conditions, suggesting that epithelial cells activate stress response pathways that induce bacteria survival [52]. In agreement with this, 290 genes were consistently upregulated in epithelial cells infected with *P. gingivalis,* highlighting those that positively regulate cell proliferation, migration, angiogenesis, inflammation, and apoptosis inhibition genes [56]. Both studies raised relevant information to determine that *P. gingivalis* inhibits epithelial cell apoptosis to generate a replicative niche in the gingival epithelium [59]. Others explored the metagenomics and transcriptional changes of human immune cells challenged with *P. gingivalis* or their LPS. Monocytes challenged with LPS showed 902 differentially expressed transcripts. These findings helped improve understanding of the *P. gingivalis*-induced immune response, identifying new transcripts and their potential association with other chronic inflammatory diseases [53]. To evaluate the role of fimbrial adhesins in innate immune responses, Arjunan et al. (2016) performed RNAseq transcriptome analyses from monocyte-derived dendritic cells (MoDCs) infected with *P. gingivalis* wild type or with a fimbrial isogenic mutant [54]. As a result, 479 differentially expressed genes were identified under different experimental conditions. Genes associated with inflammation, immune response, anti-apoptotic mechanisms, and cell proliferation, among others, showed greater positive regulation.

This evidence lets us corroborate the relevance that − omics technologies have for understanding how *P. gingivalis* rapidly adapts and responds to the diverse microenvironments explored during its pathogenesis.

## 5. *P. gingivalis* Is a Keystone Pathogen of Periodontitis

As a keystone pathogen, *P. gingivalis* disrupts the host’s immune response even at low relative abundances [60,61]. This microbe generates protection and the optimal nutritional conditions for the growth and development of pathobiont microorganisms, responsible for maintaining the destructive chronic inflammatory environment [62]. Therefore, *P. gingivalis* modulates the entire bacterial community’s composition, abundance, and adaptive fitness [63,64]. This synergistic interaction was evidenced in a mechanistic study using germ-free mice. In contrast to wild-type mice, the oral microbiota lack in germ-free mice did not induce destructive inflammation after *P. gingivalis*-inoculation into the periodontal tissues [13].

The oral microbiome comprises over 600 prevalent taxa distributed in different ecological niches. Because many of these species cannot be cultured, omics technologies are powerful tools for assessing their composition [65]. Using clinical samples, metagenomics and metatranscriptomics studies have confirmed the prominent subgingival microbial dysbiosis in individuals with periodontitis. Furthermore, they established the presence and relative abundance of *P. gingivalis* and the virulence factors upregulation associated with its pathogenesis [19,34,46,66] (Table 1 and Table 2).

The synergy interactions of the resident microbiota are fundamental in this new periodontitis model. In this sense, *Streptococcus gordonii* is considerate an accessory pathogen that contributes to increasing *P. gingivalis* virulence [7]. LuxS is a soluble mediator autoinducer 2 (AI-2) molecule of *P. gingivalis* involved in the uptake of hemin/inorganic iron and quorum sensing interspecies with *Streptococcus gordonii*. Utilizing transcriptomics was demonstrated in a LuxS-deficient strain that *P. gingivalis* fails to form microcolonies, confirming the significance of these molecules in the signaling involved in the formation and maturation of the biofilm [55]. The role of keystone bacteria has also been evaluated *in vitro*. A multispecies symbiotic biofilm (oral health) was generated in the presence or absence of *P. gingivalis* and *A. actinomycetemcomitans.* The transcriptomic analyses showed significant gene expression pattern differences when comparing both conditions. Similarly, they exhibited different gene expressions when planktonic, and biofilm conditions were compared. These findings showed a two-way modulatory effect of periodontal pathogens on multispecies heterotypic communities [57]. The keystone pathogen effect has also been evaluated in in vivo models. The ligature-induced periodontitis model in mice induces microbial dysbiosis and recreates the destructive inflammatory conditions exhibited during periodontitis in humans [67]. Sterile silk ligature alone causes alveolar bone resorption. However, when it is preincubated with *P. gingivalis* and subsequently tied to the tooth, the bone loss generated is significantly exacerbated [68]. Consistent with the systemic effects of periodontitis, oral administration of *P. gingivalis* significantly alters the gut microbiome. The metagenomic studies showed evident differences in the composition and relative abundance of the community members compared to mice that did not receive *P. gingivalis*. In addition, dysbiosis generates systemic inflammatory effects, changes in the serum metabolome, and aggravates collagen-induced arthritis [69,70,71,72]. This evidence reveals the contribution of omics disciplines to determining the keystone role of *P. gingivalis* in perturbing bacterial communities.

## 6. Proteomics and Metabolomics in *P. gingivalis* Pathogenesis

Proteomics corresponds to the study (Table 3) of the structure, function, and protein interaction on a large scale [73]. This technique has become a powerful tool for protein identification involved in host-*P. gingivalis* interactions and their relationship with other oral bacteria. For example, proteomic studies using mixed oral bacteria cultures showed that nearly 40% of *P. gingivalis* proteins can be regulated by the presence of other members of the oral microbiota, such as *Fusobacterium nucleatum*, *Streptococcus gordonii*, or *Streptococcus oralis* [74,75], suggesting an effect of the ecological community on *P. gingivalis* protein expression.

**Table 3:** Summary of metabolomics and proteomics research studies associated to this review.

Strategy	Method and Sample	Findings/Contributions	Reference
Proteomics	Cultured *P. gingivalis*, *F. nucleatum*, and *S. gordonii* mixed biofilm and *P. gingivalis* monobiofilm as control. Bacterial cells were lysed, and proteins were digested for mass spectrometry.	The proteome of the mixed biofilm differs from the monobiofilm, it exhibit a decreasent in proteins involved in cell shape and cell envelope formation, and an increasement in HmuR protein (an outer membrane receptor).	[74]
Proteomics	Cultured *P. gingivalis* (ATCC^®^33277™) and *S. oralis* (ATCC^®^9811™) mixed biofilm. Controls are monobiofilm of *P. gingivalis* and *S. oralis*. Biofilm samples were digested and then summited to shotgun proteomic analysis with LC-MS/MS.	The *P. gingivalis* proteins that increased their expression induced by the interaction with *S. oralis* were GyrB, RpoD, FimA and a probable transcriptional regulatory protein.	[75]
Proteomics	Liquid cultured *P. gingivalis* (ATCC^®^33277™, W83 and two peptidylarginine deiminase (PPAD) mutant strain) were centrifugated and the supernatant was analyzed by mass spectrometry analysis.	Analysis of the proteome and citrulinoma extracellular of *P. gingivalis* showed heterogeneity between the different isolates. Furthermore, the main virulence factors revealed different patterns in their citrullination.	[76]
Proteomics	Cultured *P. gingivalis* ATCC^®^33277™ and mutant strains. Cells were harvested, lysed, and the supernatant was subjected to mass spectrometry analysis.	Identification of 257 putative O-glycosylation sites within 145 glycoproteins of *P. gingivalis*. Demonstration for the first time the presence of the O-glycosylation system in *P. gingivalis*.	[77]
Proteomics	Cultured *P. gingivalis* (W50 strain), then cells were harvested and centrifuged, and the supernatant was filtered to obtain outer membrane vesicles (OMVs) for mass spectrometry analysis.	A total of 151 OMV proteins were identified and the most enriched proteins were LptO, IhtB and HmuY.	[78]
Proteomics	Cultured *P. gingivalis* (W50 strain) in three conditions: control, heme limitation, and heme excess conditions. Then cells were harvested and processed to obtain whole cell lysate and outer membrane vesicles separately and then mass spectrometry analyses.	The proteins most upregulated in response to heme limitation were those involved in binding and transporting heme.	[79]
Metabolomics	Tongue swabs and mouth washout samples from patients with chronic periodontal disease were analysed with proton nuclear magnetic resonance (H-NMR) to determine its metabolic status.	The metabolic state of the mouth of chronic periodontal disease patients changes in the levels of eight metabolites in comparison to healthy individuals. These metabolic changes could be used as a periodontal disease-associated process biomarker.	[80]
Metabolomics	Meditation through Gas chromatography-mass spectrometer (GC-MS) metabolite profiling of cultured human periodontal ligament fibroblast infected with *P. gingivalis* (ATCC^®^33277™).	Periodontal ligament cells (PDLSCs) experienced metabolic reprogramming due to the infection of *P. gingivalis*. These metabolic changes could be related to pro-inflammatory responses on PDLSC, showing a shift from oxidative phosporylation to glycolysis.	[81]
Metabolomics and metagenomics	Serum samples from mice submitted to an oral gabage of *P. gingivalis* (ATCC^®^33277^TM^) and sham control were analysed with Untargeted metabolomics profiling chromatographic separation and mass spectrometry (MS). Additionally, RNA extraction and metagenomic analysis was done in colon samples from the same study groups.	The analysis of the metabolites in *P. gingivalis*-administered mice demonstrated that oral administration of this periodontal pathogen could induce dysbiosis of the gut microbiota. In addition, these derived metabolites are associated with metabolic pathways and could be related to the development of metabolic disorders and the destruction of intestinal barrier function.	[82]

The identification and characterization of proteins involved in bacterial colonization and invasion is another relevant scope of *P. gingivalis* proteomics. Notable changes have been reported in the extracellular proteomic profiles and virulence factors expressed by different strains of *P. gingivalis* [76]. For instance, using mass spectrometry, alterations in the *P. gingivalis* O-glycoproteome are evidenced, determining that gingipains lacked these post-translational modifications [77]. On the other hand, a previously known marker of rheumatoid arthritis is the loss of tolerance to the presence of proteins that have undergone citrullination. This post-translational modification affects arginine residues and produces changes in the structure and function of proteins [83]. Interestingly, *P. gingivalis* contains a peptidyl-arginine deiminase (PPAD), an enzyme capable of protein citrullination, and it can modify bacterial and human proteins [76]. Moreover, the comparison of secreted protein citrullination profiles among several *P. gingivalis* clinical isolates with reference strains showed substantial differences, primarily focused on a set of six to 25 proteins, mostly composed by virulence factors. The role of these modifications remains to be clarified.

Proteomics can also help to understand the role of some cellular derivates, such as Outer membrane vesicles (OMVs). *P. gingivalis* releases several virulence factors toward the environment via the use of OMVs [78]. Studies covering the role of OMV-associated proteome in *P. gingivalis* suggest important changes according to the availability of heme, displaying an upregulation of proteins involved in heme binding and transport when this nutrient is limited [79]. Collectively, proteomic studies reveal that the *P. gingivalis* proteome changes depending on the interaction target and the biological context, offering another opportunity to understand *P. gingivalis* physiology and pathogenesis.

Another useful technique to understand the role of *P. gingivalis* in periodontitis is metabolomics (Table 3), which is the systematic study of metabolic content produced and consumed by specific cellular processes. Thus, it presents the collection of all metabolites within a particular sample [84]. Current evidence suggests that during periodontitis, a metabolic alteration in the oral cavity is induced by the colonization of pathogenic bacteria [82]. Additionally, metabolic changes are also reported in cells of the periodontal ligament (PDLSCs) under *P. gingivalis* infection, showing a metabolic reprogramming that compromises processes associated with the glycolysis, tricarboxylic acid (TCA) cycle, tryptophan, and choline metabolism [81,82]. Since gene sequence and expression profiles are essential but not sufficient to understand the mechanisms underlying *P. gingivalis* pathogenesis, proteomic and metabolomic studies have begun to gain more insights into periodontitis-related host-pathogen interactions.

## 7. Conclusions and Perspective

Nowadays, whole-genome sequencing, metagenomic, metatranscriptomic, proteomic, and metabolomic techniques have become essential strategies aimed at elucidating and understanding diverse aspects of the behavior and activity of microorganisms. As shown in the content of this mini-review, these techniques could help to understand some of the genetic principles of *P. gingivalis* role in the pathogenesis of periodontitis. In this context, data integration from different − omic disciplines offers the opportunity to understand how bacterial interactions in the periodontal biofilm could lead to undesirable host responses. This knowledge may reveal new therapeutic focuses that let us reduce its pathogenic potential. Day after day, further studies using − omics techniques reveal unknown aspects of *P. gingivalis*, opening gaps in knowledge not resolved with traditional techniques. Overall, the application of multi− omics techniques and the development of skills in data processing and integration methods are essential if we want to understand the complex system in which *P. gingivalis* interact and, in particular, if our final goal is to contribute to the control of periodontitis.

## Data Availability

No new data were created or analyzed in this study. Data sharing is not applicable to this article.
